# Wearable Atmospheric Pressure Plasma Fabrics Produced by Knitting Flexible Wire Electrodes for the Decontamination of Chemical Warfare Agents

**DOI:** 10.1038/srep40746

**Published:** 2017-01-18

**Authors:** Heesoo Jung, Jin Ah Seo, Seungki Choi

**Affiliations:** 1 Agency for Defense Development (ADD), Yuseong P.O. Box 35-5, Daejeon 305-600, Republic of Korea

## Abstract

One of the key reasons for the limited use of atmospheric pressure plasma (APP) is its inability to treat non-flat, three-dimensional (3D) surface structures, such as electronic devices and the human body, because of the rigid electrode structure required. In this study, a new APP system design—wearable APP (WAPP)—that utilizes a knitting technique to assemble flexible co-axial wire electrodes into a large-area plasma fabric is presented. The WAPP device operates in ambient air with a fully enclosed power electrode and grounded outer electrode. The plasma fabric is flexible and lightweight, and it can be scaled up for larger areas, making it attractive for wearable APP applications. Here, we report the various plasma properties of the WAPP device and successful test results showing the decontamination of toxic chemical warfare agents, namely, mustard (HD), soman (GD), and nerve (VX) agents.

Atmospheric pressure plasmas (APPs) have been actively studied since the 1990s^1^,^2^. In contrast to vacuum plasmas, which are used in confined applications, such as semiconductor processes, APPs can be discharged in open spaces (1 atm). The classification of APPs is based on their discharge structures and discharge modes, which include dielectric barrier discharge (DBD), corona jet, glow discharge, arc torch, micro-hollow cathode discharge (MHCD), and inductively coupled plasma (ICP)^1^,^2^,^3^,^4^. Plasma with the desired physical and chemical characteristics is generated by adjusting factors such as the input frequency, input voltage, input current, input waveform, and supply gas. Because of its various favorable characteristics, the application of APP to various fields, such as the biomedical, material, and energy/environmental industries, has been considered, and basic research on APPs has been actively conducted worldwide^1^,^2^,^3^,^4^,^5^,^6^,^7^,^8^,^9^,^10^,^11^,^12^,^13^,^14^,^15^,^16^.

However, more than 90% of APP research is related to small DBD or corona-jet-type plasmas that use helium or argon gas with a relatively low discharge voltage. Because a large amount of the gas supplied for discharge is consumed, facilities able to supply the gas are required, resulting in the need to simplify the system^3^,^4^,^5^,^6^,^7^,^8^,^9^,^12^. By contrast, air plasmas do not require special gases and are easy to apply in ambient environments. In APP, the major species affecting chemical/biological materials are typically attributed to radicals, reactive oxygen species (ROS; e.g., OH, O, and O_3_), reactive nitrogen species (RNS; e.g., NO and NOx), charged particles (O_2_^−^), and reactive metastables (e.g., O_2_(^1^Δ_g_)). In APP applications for which air is used instead of a discharge gas, DBD-type or torch-type electrodes are frequently utilized. However, because both the space between the electrodes and the plasma treatment sectional area are usually small, new electrode concepts that can provide large-area discharge while increasing the power efficiency are required^8^,^9^.

To overcome limitations in the treatment area, the treatment of a large flat surface area has been investigated by combining a plasma torch array and a roll-to-roll process^9^. However, in many cases, the plasma system is designed using non-flexible electrodes for generating the plasma, which restricts the volume and design of the overall system.

Currently, flexible devices such as displays, solar cells, energy storage, and sensors are important research areas^17^,^18^,^19^,^20^,^21^,^22^,^23^,^24^,^25^,^26^,^27^. In particular, there is significant interest in flexible and wearable devices for applications in flexible electronics.

In this study, we present a plasma generation device that uses a flexible plasma generation electrode assembly and a technique for generating plasma in the atmosphere without requiring any external gas. Our objective is to develop plasma textiles using flexible fabric electrodes with conducting wires that can be used to fabricate wearable garments for various applications. Therefore, our focus is on the development of a flexible electrode and an apparatus for generating plasma that can be manufactured in the form of a blanket or wearable cloth.

We also aim to provide a rapid and safe plasma detoxification and sterilization method that overcomes the various shortcomings affecting the current detoxification and sterilization systems used in civilian medical and military fields. Because of the slow rate of chemical agent degradation under environmental conditions, the ability to rapidly decontaminate solid surfaces is required for military and civil defense forces to remove the hazards introduced by these agents. Consequently, the efficient decontamination of large and complex three-dimensional (3D) surface areas using the newly developed wearable air-based APP system is demonstrated.

## Results and Discussion

### Wearable APP (WAPP)

**Figure 1:**
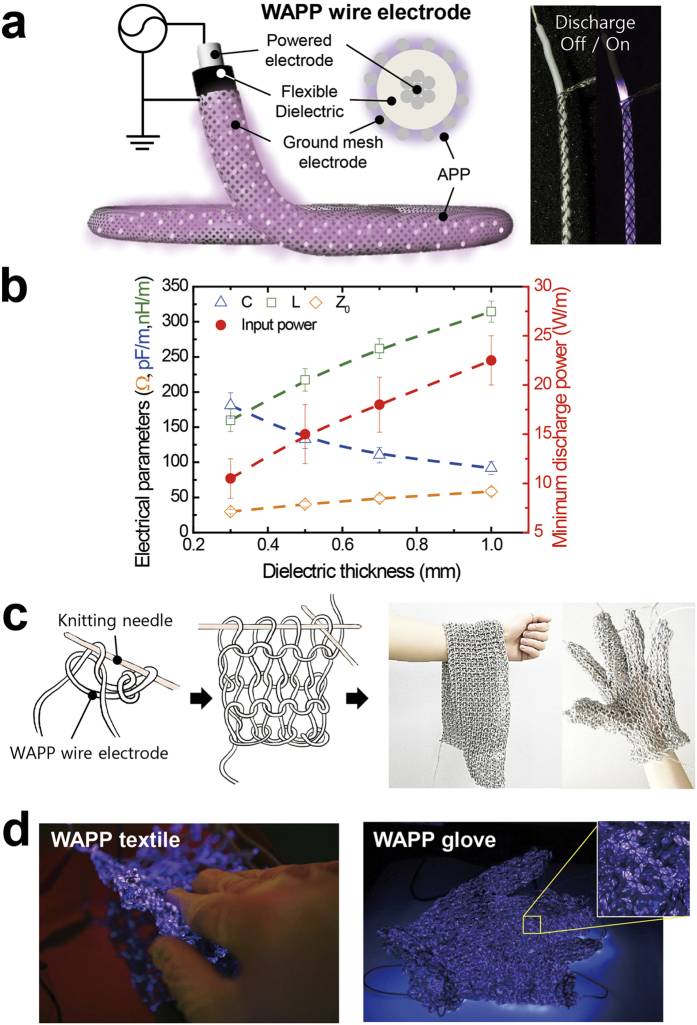
WAPP. (**a**) Schematic illustrations of the side and cross-sectional views of the WAPP single-wire electrode structure. (**b**) Dependence of the electrical parameters (inductance □, capacitance △, and impedance ◇) and minimum required input power (●) per unit length on the dielectric thickness. (**c**) Knitting method used to fabricate the large flexible plasma reactors. (**d**) Air plasma emissions of the flexible knitted textile and wearable glove.

Figure 1a presents schematic illustrations of the side and cross-sectional views of the WAPP single-wire electrode structure. The cylindrical inner electrode (i.e., the high-voltage electrode) is encased in a flexible dielectric material (heat-proof silicone). The outer electrode (i.e., the ground mesh electrode) encases the outer circumference of the dielectric material and contains numerous holes to allow the outward emission of the generated plasma. This cylindrical wire is very flexible and has a minimum outer diameter of 1.8 mm. The inner electrode comprises seven thin wires and has a diameter of 0.18 mm, and the thicknesses of the dielectric medium and outer mesh electrode are 0.3 mm and 0.35 mm, respectively. This structure appears to be very similar to that of a coaxial cable. However, the mesh braiding coverage is considerably different. In coaxial cables, increased mesh braiding coverage results in better shielding. By contrast, for plasma generation, a similar external mesh braiding coverage is suitable for an air discharge of 50% to 80%. When pulsed high voltage is applied to the inner electrode, surface DBD is generated in the open area between the inner electrode and outer mesh electrode. Therefore, the mesh braiding coverage must be varied depending on the purpose of the device. In addition, the external mesh electrodes are connected to the ground; thus, the electrode is safe to touch. Photographs of the single-wire electrode and air-discharged electrode are shown in Fig. 1a.

Figure 1b shows that the cylindrical capacitance (C), inductance (L) per unit length, and characteristic impedance (Z_0_) depend on the dielectric medium thickness (t). The capacitance and inductance per unit length of meshed wire electrode are given by 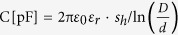 and 
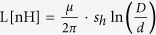
, respectively, where ε_0_ is the permittivity of free space, ε_r_ is the relative dielectric constant of the medium, s_h_ is the mesh braiding coverage, d is the outside diameter of the inner electrode, D is the inside diameter of the outer electrode, and μ is the magnetic permeability of the insulator. The silicone wire capacitance and characteristic impedance per unit length are 132.98 pF and 40.37 Ω, respectively, when a 2.2-mm wire (silicone thickness of 0.5 mm) is used. The minimum required input power for the discharge per unit length as a function of the dielectric thickness of the electrode is also shown in Fig. 1b. The average power per unit length values are 10.5 W/m, 15 W/m, 18 W/m, and 22.5 W/m when 1.8-mm, 2.2-mm, 2.4-mm, and 3.2-mm wires (corresponding to silicone thicknesses of 0.3 mm, 0.5 mm, 0.7 mm, and 1.0 mm) are used, respectively.

In this work, the plasmas were generated with a low-frequency pulse power at a frequency of 10–40 kHz in ambient air with a nominal input power (power consumption) of approximately 50–200 W for a plasma area of 100 mm × 100 mm using our 2.2-mm WAPP wire electrode. Logically, the power must be increased to discharge the plasma over a long, large area. The circuit characteristics are more strongly affected by the length of the wire electrode than by the knitting pattern. However, if the wire length in a given fabric is very long, the input power depends not only on the wire length but also on its geometric structure. Therefore, we plan to further optimize the flexible nanowire geometric structure for low-power air discharge in the future. In this study, we focus on the development of a new method for producing a knitted flexible source using our WAPP wire electrode and its potential applications.

Figure 1c illustrates the knitting method used to fabricate large flexible plasma reactors, such as hand-knitted towels (550 mm × 150 mm, C = 6.24 nF, total wire length = 48 m) and gloves (200 mm × 150 mm, C = 2.7 nF, total wire length = 20.4 m) using 2.2-mm wire. As shown in Fig. 1c, the wearable plasma electrode system can be easily incorporated into the textile weaving process to achieve high throughput. Low-frequency power was provided to the knitted electrode using a power system consisting of a 15-kHz pulse or sine wave power supply. The measured peak-to-peak voltage was 7 kV for discharge, and the total power consumption was in the range of 250–500 W.

Figure 1d presents photographs of the flexible knitted textile and wearable glove air plasma emission taken using a digital single-lens reflex camera (Nikon D810). It should be noted that a relatively long exposure time was used to capture the plasma images because most of the emissions were in the near-UV range of 300–400 nm. The images were obtained according to International Organization for Standardization (ISO) 8000 requirements, and the exposure time was 1.3 s. When the total input power was fixed at 300 W throughout the discharge, the color of the air plasma was bright violet to the naked eye. Because the discharge electrode is safe to touch, we can treat complex 3D surfaces using this wearable electrode system. Therefore, we believe that this system will be very useful for the destruction of contaminants on surfaces in toxic chemical or for bio/medical applications.

### Decontamination test chamber and real chemical agents used in the experiments

**Figure 2:**
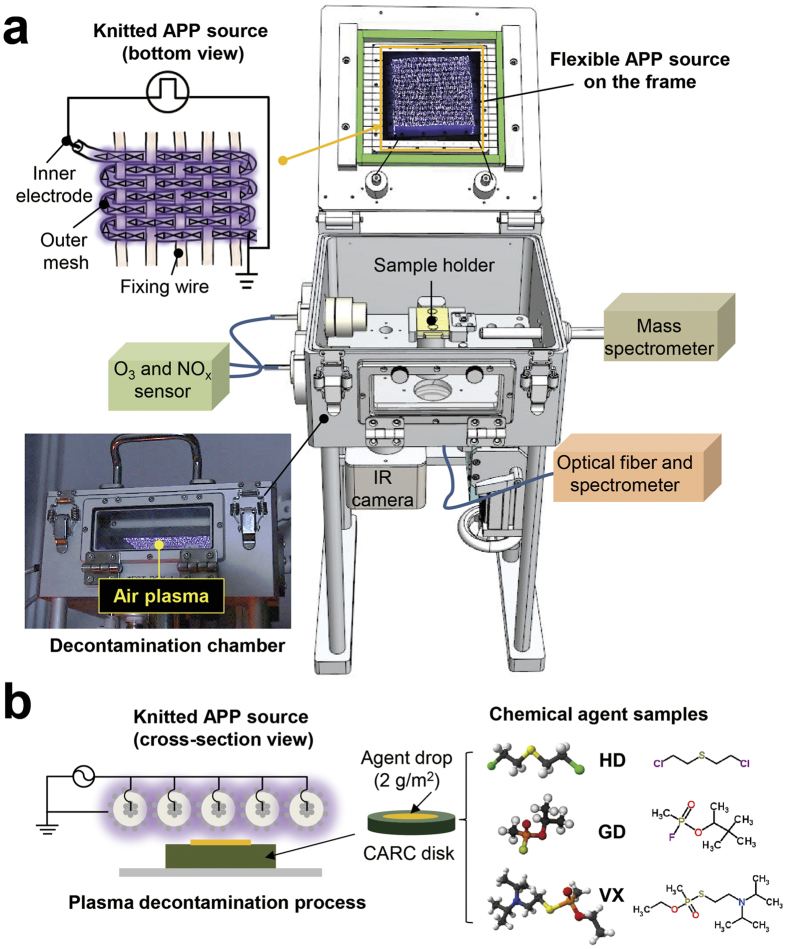
Knitted APP source, decontamination test chamber, diagnostic tools, schematic of the experimental set-up, and the treated chemical agents. (**a**) Knitted APP source (bottom view), decontamination chamber with the plasma source, diagnostic sensors, and a photograph of the air plasma discharge in the decontamination chamber (tilted view). (**b**) Plasma decontamination process using the knitted APP source (cross-sectional view) and CARC disk with real samples of chemical agents (HD, GD, and VX).

The plasma diagnostic and decontamination process was conducted in an enclosed test chamber (5,000 cm^3^) using flexible knitted electrodes (100 mm × 100 mm, C = 1.3 nF, dielectric thickness = 0.5 mm, total wire length = 10 m). To maintain a constant distance between the samples, the samples were fixed to flexible knitted electrodes on a frame. The diagnostic modules, plasma emission fiber, and temperature, ozone, and nitrogen oxide compound sensors were controlled and monitored using the NI LabVIEW system (Fig. 2a). In the diagnostic and decontamination process, APP was generated using 10% duty pulse waves of 9 kV and 10 kHz. The total input power was fixed at 150 W throughout the experiment. The plasma was discharged in ambient air without any external gas being supplied.

Figure 2b shows the side of the decontamination test chamber and the flexible air plasma source used in the experiment. The chemical agent samples were 2-2′-dichloro-diethyl sulfide (HD), O-pinacolyl methylphosphonofluoridate (GD), and O-ethyl S-[2-(diisopropylamino)ethyl] methylphosphonothioate (VX); these agents can persist in the environment for several weeks following their release^28^,^29^,^30^,^31^,^32^. VX can remain on surface materials, whereas some of the other agents can re-evaporate into the air, threatening both the environment and humans^28^,^29^,^30^,^31^,^32^. All chemical agent samples were coated at the same initial contamination density (2 g/m^2^) on a disk resistant to chemical agents. The size of the gap between the samples and the plasma surface was maintained at 2 mm.

### WAPP characteristics in the decontamination chamber

**Figure 3:**
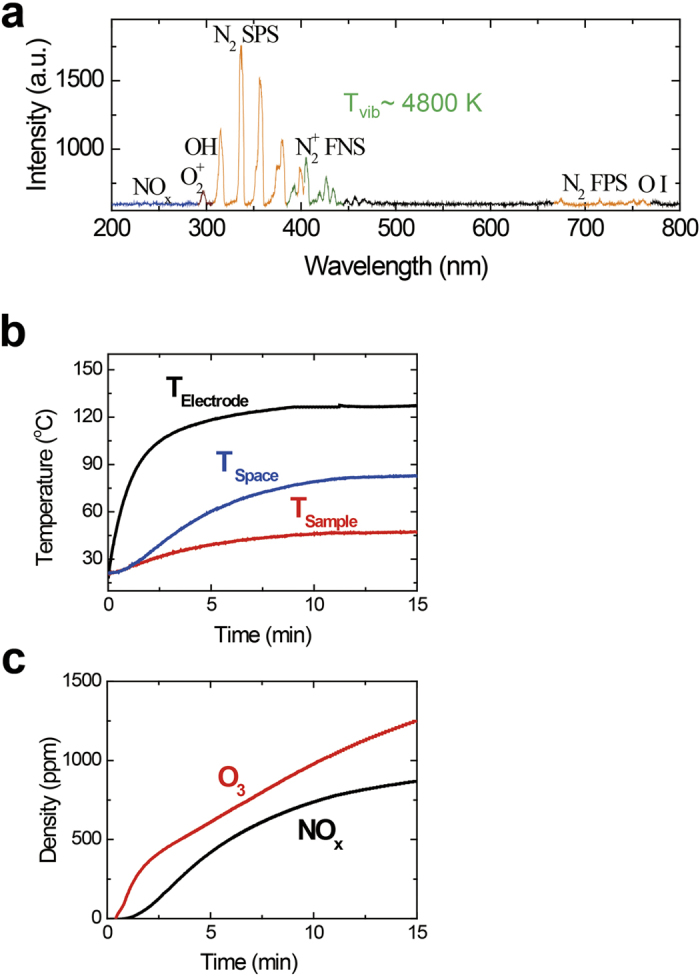
WAPP characteristics in the decontamination chamber. (**a**) Emission spectrum of the air plasma and the vibrational temperature based on the N_2_ second positive system. (**b**) Electrode, space between the electrode and the sample, and the sample temperature profile determined by the IR camera. (**c**) O_3_ and NO_x_ density profiles in the enclosed decontamination chamber during plasma discharge.

Figure 3a shows the emission spectrum of the air plasma. The N_2_ second positive system (C^3^Π_u_ → B^3^Π_g_, 330–425 nm) in the emission spectrum was used to estimate the vibrational temperature (T_vib_) in the plasma plume, and was estimated to be approximately 4800 K. The intensities of the vibrational band heads facilitated the measurement of T_vib_ using the Boltzmann plot method^9^,^10^,^13^. In addition, NO_x_, O_2_^+^, N_2_^+^, and OH molecular bands corresponding to nitrogen, oxygen, and water vapor in the ambient air were observed.

The size of the gap between the powered electrode and the treatment sample was fixed at 2 mm to allow for uniform surface discharge generation over the treated substrate. The surface temperature of the electrode was increased to 125 °C at an input power of 150 W during the 15-minute plasma treatment, the gas temperature between the electrode and the sample was less than 80 °C, and the temperature of the treated sample was less than 40 °C, as shown in Fig. 3b. These temperatures were measured using a fiber-based thermocouple and infrared (IR) camera system. For comparison, the decomposition temperatures of HD, GD, and VX are 217 °C, 130 °C, and 150 °C, respectively^29^,^30^,^31^,^32^. Therefore, the thermal effects of the system components do not play a major role in the removal of the agents from the surface. In a previous paper that compared the results of plasma and hot gas^33^, the plasma exposure results for a VX simulant indicated that removal began at temperatures as low as 50 °C, whereas for hot gas, removal was not observed until 120 °C. Furthermore, hot gas results in only the evaporation of the sample and not chemical conversion, as observed for the mustard simulant.

The concentration profiles of the long-lived ozone and nitrogen oxide compounds in the afterglow region over time, which were measured using O_3_ and NO_x_ electrochemical sensors placed on the side of the test chamber (Fig. 2a), are shown in Fig. 3c. No gas flow was supplied to the decontamination chamber during the plasma treatment to prevent an accidental release of chemical agents. The densities of O_3_ and NO_x_ increased gradually to 1300 ppm and 900 ppm, respectively, during the 15-minute plasma treatment. These gradual increases in the O_3_ and NO_x_ concentrations are attributable to the long lifetimes of these species in ambient air.

### Decontamination results on chemical agent-resistant coating (CARC) surfaces: Residual peak results

**Figure 4:**
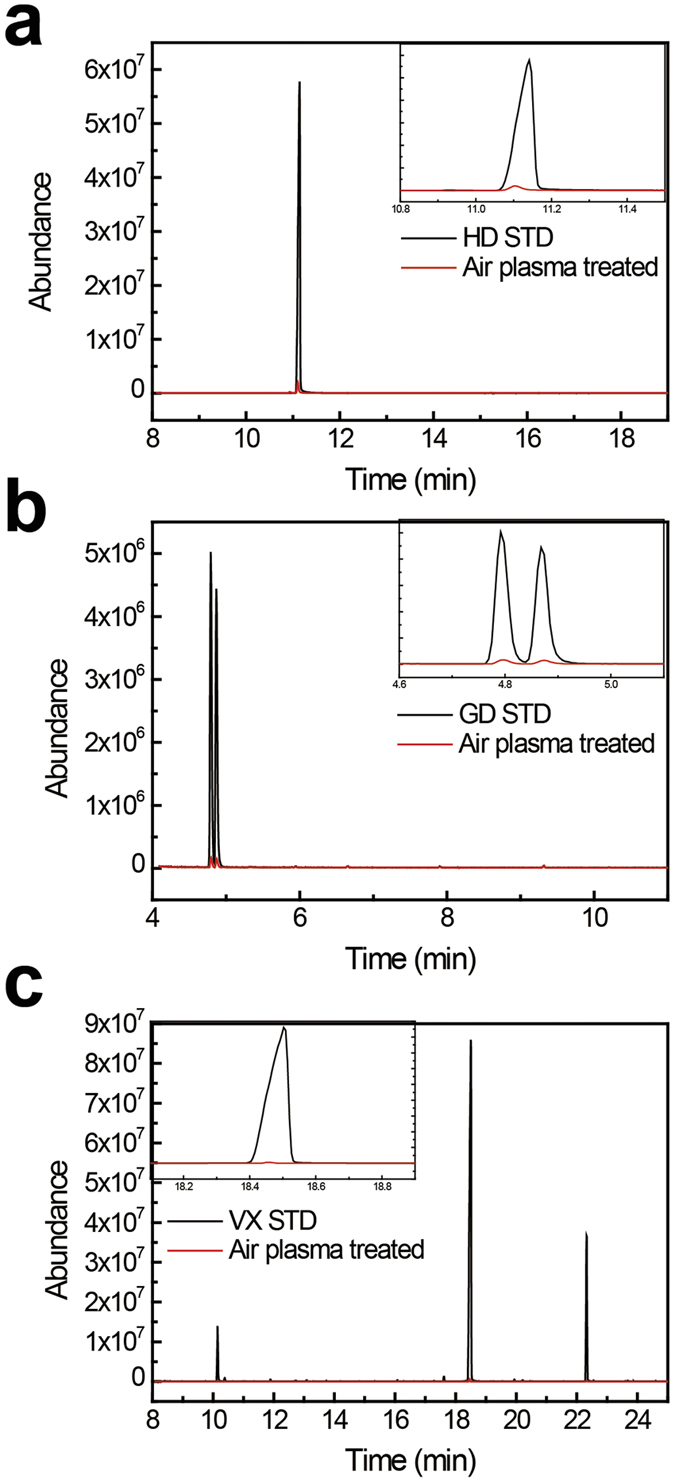
Decontamination results on the CARC surface. GC-MS spectra displaying the unique standard and residual peaks of (**a**) mustard (HD) 97%, (**b**) soman (GD) 97%, and (**c**) nerve agent (VX) 98% after APP treatment.

To investigate the effectiveness of the flexible APP for the decontamination of chemical agents on a solid surface, CARC disks contaminated with the agents were exposed to the plasma in the decontamination test chamber, and an untreated sample was also tested. Then, the residual concentrations of the agents on the surfaces were compared. The residual concentrations of the agents on the CARC disk were measured using gas chromatography mass spectrometry (GC-MS). Figure 4a,b and c present the results of the residual peaks of the mustard (HD), soman (GD), and nerve agent (VX), respectively, on the treated CARC surfaces. When APP treatment was applied, the intensities of the HD, GD, and VX residual peaks significantly decreased during the plasma discharge process relative to the untreated sample. Ninety-seven percent of HD and GD and 98% of VX were removed after approximately 15 min of exposure to the air plasma. Because of the effect of agent evaporation and decontamination by the plasma, the surface residue of the agents was reduced.

### Decontamination results in the test chamber: Mass spectra of the vaporized agent gases

**Figure 5:**
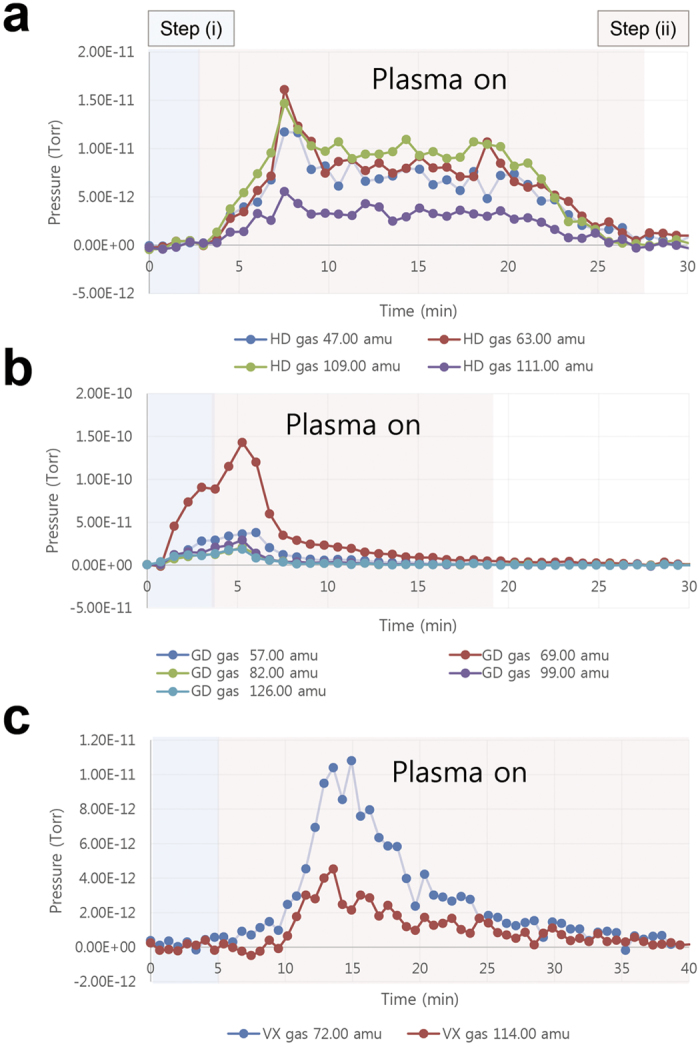
Decontamination results in the test chamber space. Mass spectra and major characteristic ions of the vaporized agent gases (**a**) HD, (**b**) GD, and (**c**) VX during APP treatment obtained using the online mass spectrometer system.

The vaporization profiles of the chemical agents during the plasma treatment were measured using an online mass spectrometer. The results are shown in Fig. 5. When the agents were placed in the chamber, their mass peaks exhibited the characteristics shown in step (i). The increasing slope corresponded to the evaporation characteristics of the agents. The GD and HD peaks increased more rapidly than the VX peaks because of the characteristic volatility of those agents. In other words, the vapor pressures of GD, HD, and VX are 13 Pa, 53 Pa, and 0.09 Pa, respectively, at 25 °C^29^,^30^,^31^,^32^. Moreover, VX is known to be a persistent chemical warfare agent and is very difficult to remove. When the decontamination process with plasma discharge began in step (ii), several mass peaks of the agents increased slightly. However, the rate of change in the masses gradually decreased. Finally, the level of agents reached the lowest point, indicating that the vaporized gases of the agents were also decontaminated by the flexible APP in the decontamination chamber. The vaporized HD, GD, and VX agents were removed after approximately 25 min, 15 min, and 35 min, respectively, of exposure to the air plasma.

### Destruction energy and plasma species

Previous reports have suggested that the selective hydrolysis energies of the phosphorothioate (PS) bond of VX, the phosphorus pentafluoride (PF) bond of GD, and the chloroalkane bond of HD are 123 kJ/mol, 83 kJ/mol, and 43 kJ/mol, respectively^34^,^35^. The flexible knitted plasmas generate non-thermal plasma by releasing electrons with high kinetic energies in the range of 290 to 580 kJ/mol by creating an electrical discharge between pairs of electrodes and by accelerating the electrons in the electrical field^16^. Therefore, the energy of the electrons is exploited to activate atmospheric oxygen and nitrogen, resulting in the generation of ROS and RNS that should be capable of breaking toxic chemical bonds via oxidation. To generate ozone, the plasma discharge initially breaks down a molecule of dioxygen into atomic oxygen. The significant amount of energy required for this process (approximately 500 kJ/mol) comes from an electrical discharge^15^. Although the input energy is much lower, the emergence of ozone is inevitable because of the working principle (the energy input conforms to a Boltzmann distribution). Given the relatively long life of ozone, its release outside the plasma zone is plausible. Therefore, the ROS in the surface plasma region can effectively remove organic binders via oxidation because of their high potential energies. These highly reactive oxygen atoms can also eliminate chemical agents on the CARC surface. Furthermore, the low gas temperature of the APP treatment protects the CARC surface from thermal damage.

### Possible explanation for the detoxification achieved using plasma

One possible explanation for detoxification achieved using plasma is the effective energy transfer between the vibrationally excited O_2_ and N_2_ molecules and the chemical agents. Past studies have shown that APP can generate vibrationally excited molecules with vibrational temperatures of up to 6000 °C, far exceeding the rotational and kinetic gas temperature of approximately 190 °C^13^. If the energy of these highly excited molecules in the plasma could be transferred to the chemical agents, the lattice atoms could rearrange themselves, resulting in dissociated molecular structures.

In addition, the increased hydrophilic properties of the APP-treated sample surface can be explained by the presence of OH radicals in the plasma. Because the APP system operates in ambient air, a small amount of water vapor can enter the plasma and form OH radicals via electron impact dissociation^14^,^15^,^16^. Separately, the reactive radicals produced by the APP system, such as O_3_ and NO_x_, may play a role because of their relatively long lifetimes in ambient air^3^,^4^. We plan to conduct further investigations and examine the roles of these species in the decontamination of chemical agents in future studies.

In summary, we presented a method for fabricating flexible, wearable APP devices via a single-wire knitting technique. Using this method, a uniform plasma with a large area can be obtained. To the best of our knowledge, this flexible electrode-knitting technique is the first successful method for creating wearable plasma sources and demonstrates the full capacity of the APP system for various applications. In addition, this wearable plasma source can discharge into ambient air without requiring any external gas. The experimental results show that the wearable air plasma can completely decontaminate toxic chemical compounds. Furthermore, this APP process removes chemical agents while preserving the sample surface. Ninety-seven percent or more of the chemical agents (HD, GD, and VX) were removed from the surface after 15 min of exposure to the air plasma (150 W). We anticipate the results of this study will support the development of a flexible electrode with flexible dielectric wires that can be used to make wearable garments for various 3D surface treatment applications, such as 3D wound healing and 3D food and toxic surface decontamination. Therefore, this wearable APP technology has potential applications in the areas of biomedical, chemical, and materials engineering. We are currently attempting to optimize a flexible nanowire fabric structure for low-power air discharge.

## Methods

### Plasma diagnostics and gas sensing

The wire electrode capacitances were directly measured using an inductance, capacitance, and resistance (LCR) meter (Agilent Technologies, HP 4263B, USA). Plasma optical emission spectroscopy (OES; Ocean Optics, high-resolution spectrometer HR2000+, USA) was performed in the 200 to 800 nm range to identify the atomic lines and molecular bands. An IR camera (Optris P160, Germany) was used to measure the electrode and sample temperatures in the range of 0 to 250 °C. Relative humidity sensors were placed inside the chamber to monitor the internal temperature and humidity in real time. Ozone and NO_x_ electrochemical sensors (ATi, Pittsburgh, PA, USA) were also placed inside the chamber to provide real-time readings on the concentrations of ozone and nitrogen oxide compounds. The O_3_ and NO_x_ gases diffuse into the sensor and travel through the back of the porous membrane to the working electrode, where they are oxidized or reduced. This electrochemical reaction results in an electric current that passes through the external circuit. The output from the sensor is linearly proportional to the gas concentration.

### Sample preparation and decontamination

*Caution: VX, HD, and GD are highly toxic chemical agents, and care must be taken to prevent exposure to the liquid and vapor. The following procedure should be performed only by trained personnel using applicable safety procedures. The agents were obtained from the Chemical Analysis Test and Research Lab, Agency for Defense Development (ADD) with a purity of* >*95%, as determined by GC-MS analysis*.

The CARC disks (24 mm in diameter) were washed in a laboratory detergent; rinsed extensively with deionized water, hexane, and ethanol; and blown dry using nitrogen gas.

Single drops of VX (0.9 μl), GD (0.9 μl), and HD (0.7 μl) (2 g/m^2^) were loaded onto the disks. All reactions were performed at room temperature (22–28 °C). The relative humidity was maintained at less than 50% during the decontamination process. The prepared agents loaded on the CARC disk were mounted onto a quartz plate under the flexible knitted electrode in the middle of the decontamination test chamber.

### Surface residual agent analysis

The plasma-treated CARC disks were removed from the decontamination test chamber after each run and placed into 25-mL glass vials. Then, 10 mL of ethyl acetate was added to extract any residual agents and/or decomposition products for GC-MS analysis. The GC-MS system used was an Agilent 6890N Gas Chromatograph (Santa Clara, CA, USA) with an HP-5MS (length: 30 m; id: 0.25 mm; film thickness: 0.25 μm) capillary column and an Agilent 5975C mass selective detector. The oven set to 45 °C for 2 min, and then, the temperature was increased to 280 °C at 10 °C/min. One microliter of each sample was injected into a split/splitless injection port at 250 °C. The split vent was turned on at 1.20 min with a purge flow of 50 mL/min of helium. The mass spectrometer was operated in electron ionization (EI; scanned from 40 to 550 amu) and chemical ionization (CI; scanned from 60 to 550 amu) modes.

### Real-time analysis of the vaporized agent gases

The masses of the agent gases vaporized from the surface of the disk in the test chamber were measured using a real-time QIC gas analysis system (Mass spectrometer, Hiden, HPR-20 QIC advanced, U.K.). The quadrupole mass spectrometer (Triple Filter HAL/3F 201 RC) was used in the range of 1 to 40 amu (start, 10^−5^ torr) in Faraday detecting mode and from 41 to 130 amu (start, 10^−10^ torr) in secondary electron multiplier (SEM) detecting mode. The QIC capillary gas-sampling inlet was used for continuous sampling, and the sample pressure ranged from 100 mbar to 2 bar. Gas sampling was performed continuously, and gas sample flow rate was 1 mL/min. The capillary inlet was operated at a temperature of 120 °C. The mass spectra of the HD, GD, and VX peaks included the following major characteristic ions: amu_(HD)_ = 47, 63, 109, and 111; amu_(GD)_ = 57, 69, 82, 99, and 126; and amu_(VX)_ = 72 and 114.

## Additional Information

**How to cite this article**: Jung, H. *et al*. Wearable Atmospheric Pressure Plasma Fabrics Produced by Knitting Flexible Wire Electrodes for the Decontamination of Chemical Warfare Agents. *Sci. Rep.* **7**, 40746; doi: 10.1038/srep40746 (2017).

**Publisher's note:** Springer Nature remains neutral with regard to jurisdictional claims in published maps and institutional affiliations.
